# Diet quality from mid to late life and its association with physical frailty in late life in a cohort of Chinese adults

**DOI:** 10.1186/s12937-024-00964-y

**Published:** 2024-06-04

**Authors:** Jun S. Lai, Kevin Y. Chua, Huiqi Li, Woon-Puay Koh

**Affiliations:** 1 Singapore Institute for Clinical Sciences, Agency for Science Technology and Research, Brenner Centre for Molecular Medicine, 30 Medical Drive, Singapore, 117609 Singapore; 2 Integrative Sciences and Engineering Programme, NUS Graduate School, National University of Singapore, Singapore, 119077 Singapore; 3 Healthy Longevity Translational Research Programme, Yong Loo Lin School of Medicine, National University of Singapore, Singapore, 119228 Singapore

**Keywords:** Diet quality, Dash, Mid-life, Frailty, Late-life, Chinese

## Abstract

**Background:**

It is unclear if improving diet quality after midlife could reduce the risk of physical frailty at late life. We aimed to associate changes in diet quality after midlife with physical frailty at late life.

**Methods:**

Diet quality in 12,580 participants from the Singapore Chinese Health Study was assessed with the Dietary Approaches to Stop Hypertension (DASH) scores at baseline (1993–1998; mean age 53 years) and follow-up 3 (2014–2016; mean age 73 years). Physical frailty was assessed using the modified Cardiovascular Health Study phenotype at follow-up 3. Multivariable logistic regressions examined associations between DASH scores and physical frailty.

**Results:**

Comparing participants in extreme quartiles of DASH scores, the odds ratios (OR) [95% confidence interval (CI)] for physical frailty were 0.85 (0.73,0.99) at baseline and 0.49 (0.41, 0.58) at follow-up 3. Compared to participants with consistently low DASH scores, participants with consistently high scores (OR 0.74, 95% CI: 0.59, 0.94) and those with > 10% increase in scores (OR 0.78, 95% CI: 0.64, 0.95) had lower odds of frailty. Compared to those in the lowest DASH tertiles at both time-points, significantly lower odds of physical frailty were observed in those who were in the highest DASH tertiles at both time points [0.59 (0.48, 0.73)], and in those who improved their scores from the lowest [0.68 (0.51, 0.91)] or second tertile at baseline [0.61 (0.48, 0.76)] to the highest tertile at follow-up 3.

**Conclusions:**

Maintaining a high diet quality or a substantial improvement in diet quality after midlife could lower the risk of physical frailty at late life.

**Supplementary Information:**

The online version contains supplementary material available at 10.1186/s12937-024-00964-y.

## Background

Globally, the rise in human life expectancy has resulted in rapid population ageing. It is projected that by 2050, all regions of the world, with the exception of Africa, will have 25% or more of their populations being aged 60 years and older [1]. Accompanying an ageing population is a rise in age-related physical frailty, which is characterized by diminished strength and endurance, and reduced physiologic function [2]. Physical frailty has been shown to increase risk of disability, hospitalization, and mortality, thus placing a huge burden on affected individuals, their families, the health care system and society [3].

A review of evidence from intervention studies confirmed the view that physical frailty is a reversible condition through lifestyle changes [4]. However, the majority of dietary interventions to reverse or delay onset or progression of physical frailty were conducted in frail and pre-frail community-dwelling older adults, those in hospital or in long term institutional care, or those with chronic conditions such as type 2 diabetes [4–9]. Hence, it is crucial to demonstrate associations of overall dietary patterns and physical frailty in community-dwelling adults who are not yet frail or pre-frail, in order to establish evidence for frailty prevention later in life in the general population.

A “healthy” or high-quality dietary pattern is generally characterised by higher consumption of fruits, vegetables, and whole grains, and lower consumption of red and processed meat, and are exemplified by the Mediterranean diet, the Dietary Approaches to Stop Hypertension (DASH) diet, and plant-based diets [10]. Studies have shown that adherence to these aforementioned dietary patterns were associated with lower risks of frailty [10]. However, most of these studies are cross-sectional or short term (< 5 years) prospective studies, which assessed both dietary patterns and physical frailty at late life [10].

Understanding whether adopting a “healthy” or high-quality dietary pattern at midlife (aged 45–65 years) as well as how improvement in dietary patterns after midlife may lower the risk of physical frailty in late life (aged ≥ 65 years) can help determine whether dietary interventions to prevent physical frailty need to start as early as midlife, and whether there is still value in intervening after midlife. A few studies have shown a “healthy” or high-quality dietary pattern at midlife to prospectively associate with a lower risk of physical frailty at late life [11–16]. However, these studies did not examine changes in diet over time. To date, only two studies have demonstrated that maintenance of a high-quality diet in the long term (6–20 years) from midlife was associated with lower risks of physical frailty in late life but both studies were conducted in Western populations [17, 18], who have different dietary intakes and practices from Asian populations [19]. Therefore, the present study aimed to fill this gap by examining the association of changes in diet quality from mid to late life, with the risk of physical frailty at late life in a cohort of Chinese adults living in Singapore.

## Methods

### Study population

Data for the present analysis are drawn from the Singapore Chinese Health Study (SCHS) – an ongoing population-based cohort study in Singapore that recruited 63,257 Chinese men and women aged 45–74 years between April 1993 and December 1998 [20]. Participants were citizens or permanent residents, of Chinese ethnicity and belonging to the two major dialect groups (Hokkien and Cantonese) and residing in public housing during the recruitment period. Further details on the SCHS have been published elsewhere [20]. Surviving participants were re-contacted for three follow-up interviews in 1999–2004 (follow-up 1), 2006–2010 (follow-up 2), and 2014–2017 (follow-up 3), respectively. The present analysis included all participants who completed dietary assessments at baseline and at follow-up 3, as well as assessment of physical frailty criteria (weight loss, exhaustion, slowness, and weakness) at follow-up 3. All procedures in the SCHS were approved by the Institutional Review Board of the National University of Singapore. All study participants gave written informed consent. The Strengthening the Reporting of Observational Studies in Epidemiology-Nutritional Epidemiology (STROBE-nut) checklist was used for the reporting of this study (Additional File 1).

### Dietary intakes at baseline and follow-up 3

At baseline, dietary intake in the past one year was assessed using an interviewer-administered, 165-item, semi-quantitative food frequency questionnaire (FFQ), which had been validated in a subset of the cohort against 24-hour recalls and repeat administration of the FFQ [20]. Participants were asked to indicate their frequency of consuming each food item from eight frequency options, ranging from “never or hardly ever” to “two or more times a day,” as well as the usual amount consumed from three portion size images. For non-alcoholic beverages, participants indicated their consumption frequency of a standard serving (one glass/cup – 237 ml) from nine frequency options ranging from “never or hardly ever” to “six or more times a day.” Energy intake per day (kcal) was estimated using the Singapore Food Composition Database specifically developed for our cohort.

At follow-up 3, dietary intake in the past one year was assessed using a modified dietary screener administered by trained interviewers [21]. The dietary screener assessed consumption of 21 food and beverage items, and was designed to capture sufficient information to assess diet quality according to international healthy eating indices or scores such as the Dietary Approaches to Stop Hypertension (DASH) diet score [22]. It has shown reasonable validity and reproducibility against a comprehensive 163-item long FFQ in assessing food groups represented in these indices or scores in the local population [21]. Participants were required to indicate their frequency of consuming one standard serving (based on local serving sizes) of each item from 10 frequency options ranging from “never or rarely” to “6 + a day.” Additional questions on the brand and name of breakfast cereals consumed were used to determine the wholegrain content of their breakfast cereals.

### Diet quality at baseline and follow-up 3

Diet quality at baseline and follow-up 3 were scored by measuring adherence to a modified DASH diet. The original score included eight components and each component was assigned one to five points according to participants’ quintile of intake, whereby five points were given for the highest quintile of whole grains, vegetables, fruits, nuts and legumes, and low-fat dairy intakes, and the lowest quintile of sugar-sweetened beverages and fruit juice, red and processed meat, and sodium intake (reverse-scoring) [22]. We used total dairy intake as a surrogate for the calculation of the low-fat dairy component as the information collected on dairy products at both baseline and follow-up 3 did not distinguish between full- and low-fat. Additionally, we excluded the sodium component from the calculation of total DASH score as sodium intake was not adequately captured by our dietary instruments at both time points; hence, our modified DASH score consisted of seven components, and only had a maximum score of 35 points. Further details on the scoring method have been published elsewhere [23].

### Physical frailty at follow-up 3

Physical frailty was assessed using a modified version of the Cardiovascular Health Study (CHS) frailty phenotype that included weight loss, exhaustion, slowness, and weakness measured at follow-up 3 [2, 24, 25]. Although the original frailty phenotype was defined using five criteria [2], we were not able to include the criterion of low activity in our cohort as this information was not collected at follow-up 3 [24, 25]. Weight loss was defined as losing 10% or more of their self-reported body weight since follow-up 2 interviews conducted between 2006 and 2010, which were about 7.3 ± 1.0 (mean ± SD) years before follow-up 3. Exhaustion was defined as answering ‘No’ to the question “Do you feel full of energy?”. Slowness was defined as being in the lowest sex-specific quintile timing for the timed up-and-go (TUG) test, whilst weakness was defined as being in the lowest sex-specific quintile for handgrip strength test. Participants who met ≥ 2 of these criteria were classified as being physically frail.

### Covariates

At baseline, self-reported information on gender, dialect groups (Hokkien or Cantonese), highest educational level attained (no formal education, primary school, secondary school and above), marital status (married, separated/divorced, widowed, never married), cigarette smoking (never, former, current), alcohol drinking (none, monthly, weekly, daily), weekly activities [hours per week spent on moderate activities (none, 0.5-1, 2–3, 4–6, 7 + hours), strenuous sports (none, 0.5-1, 2–3, 4 + hours), and vigorous work (none, 0.5-3, 4–6, 7 + hours)], sleep duration per day (≤ 5, 6, 7, 8, 9 + hours), height, weight, and history of physician-diagnosed cancer, hypertension, diabetes, coronary artery disease, stroke or diabetes were obtained by trained interviewers using a standardized questionnaire. Body mass index (BMI, kg/m^2^) was calculated based on self-reported weight in kilograms divided by height in meters squared and categorized according to WHO recommendations for weight categories in Asians: <18.5, 18.5–22.9, 23.0–27.4, ≥ 27.5 [26].

Using the same questionnaire, weekly activities and alcohol drinking were re-assessed at follow-up 2, while marital status, cigarette smoking, sleep duration, height, weight, and physician-diagnosed cancer, hypertension, diabetes, coronary artery disease, stroke or diabetes were re-assessed at follow-up 3. Participants’ status of cancer, hypertension, coronary artery disease, stroke, and diabetes at follow-up 3 were defined as none (never reported at baseline and follow-up 3), reported at baseline, or new cases at follow-up 3.

### Statistical analyses

Changes in diet quality were computed by subtracting DASH score at follow-up 3 from DASH score at baseline. Participants were then categorized into large decrease (> 10% decrease; equivalent to <-3.5 points), small decrease (3–10% decrease; <-1 to ≥-3.5 points; ), stable (< 3% change; ±1 point), small increase (3–10% increase; >1 to ≤ 3.5 points), and large increase (> 10% increase; >3.5 points) [23]. The percentage cut-offs used were based on the definition from a US-based study on changes in diet quality [27]. Participants in the stable group were further divided into those with “consistently low” or “consistently high” scores if their DASH scores at both time points were below or above 21 points (median score of the study sample) [23]. The characteristics of participants at baseline according to changes in diet quality were compared using one-way ANOVA for continuous variables, and chi-squared test for categorical variables.

We first examined the associations of baseline and follow-up 3 DASH score quartiles with physical frailty at follow-up 3, respectively, using multivariable logistic regressions. Tests for linear trends were conducted using the median value of each quartile of the respective DASH score. The models were first adjusted for age at physical tests, gender, dialect group, and highest level of education attained (Model 1), and then further adjusted for the following covariates at baseline (Model 2a) or at follow-up 2 and 3 visits (Model 2b): physician-diagnosed hypertension, coronary artery disease, stroke, diabetes, cancer, alcohol consumption, cigarette smoking, BMI categories, sleep duration per day, amount of moderate activities, strenuous sports and vigorous work per week, and daily energy intake (only at baseline). The associations between changes in diet quality and physical frailty were also examined using multivariable logistic regression adjusted for age at physical tests, gender, dialect group, and highest level of education attained (Model 1), and for physician-diagnosed hypertension, coronary artery disease, stroke, diabetes, cancer, alcohol consumption, cigarette smoking, BMI categories, sleep duration per day, amount of moderate activities, strenuous sports and vigorous work per week, and daily energy intake at baseline (Model 2) and/or at follow-up 2 and 3 visits (Model 3). Additionally, we performed a joint analysis of DASH score tertiles at baseline and follow-up 3 in relation to physical frailty at follow-up 3 and adjusting for the same covariates.

The associations of baseline and follow-up 3 DASH score quartiles, as well as changes in diet quality, with the odds of physical frailty were repeated with each individual criterion of physical frailty phenotype (weight loss, exhaustion, slowness, weakness). Stratified analyses by age group at baseline (< 55, ≥ 55 years) and gender were performed for the associations between changes in diet quality and physical frailty. Test for interactions were performed by adding an interaction term in Model 2. Finally, the analysis associating changes in diet quality to physical frailty was repeated excluding participants who met either of two criteria at the baseline interviews: (1) those who were 60 years old and above; (2) those who had a baseline history of hypertension, cardiovascular disease, diabetes, or cancer.

All statistical analyses were conducted using Stata/SE 17.0 software (StataCorp LLC, College Station, TX, USA). A significance level of 0.05 was used for the statistical tests.

## Results

A total of 12,580 participants who completed the FFQ at baseline and the dietary screener at follow-up 3, and had complete data for physical frailty assessments at follow-up 3 were included in the present analysis (Additional File 2). At baseline, participants who had “consistently high” DASH scores were younger, more likely to be women and to have attained higher educational level compared to their counterparts with lower DASH scores. Conversely, they were less likely to consume alcohol frequently (weekly/daily) or smoke, but more likely to engage in physical activities. They were also less likely to have a history of coronary artery disease (relative to “consistently low”), but more likely to have a history of diabetes and cancer at baseline (Table 1). Similar participant characteristics were observed at follow-up 3, but these individuals were more likely to have history of coronary artery disease, but less likely to have stroke and diabetes (relative to “consistently low”) at follow-up 3 (Additional File 3).

**Table 1:** Characteristics of study participants at baseline (unless otherwise specified) by changes in DASH^1^ scores^2,3^

	Consistently low (< 3% change and ≤ median DASH at both timepoints)	Large decrease (> 10% decrease)	Small decrease (3–10% decrease)	Small increase (3–10% increase)	Large increase (> 10% increase)	Consistently high (< 3% change and > median DASH at both timepoints)
	*n* = 1512	*n* = 3377	*n* = 1820	*n* = 1659	*n* = 2699	*n* = 1513
DASH score at baseline	17.6 ± 2.3	24.1 ± 3.9	21.9 ± 4.0	19.5 ± 3.8	17.8 ± 3.5	23.9 ± 2.4
DASH score at follow-up 3	17.6 ± 2.3	17.5 ± 3.8	19.4 ± 4.0	21.9 ± 3.8	24.2 ± 3.6	23.9 ± 2.4
Age at baseline, years	52.8 ± 5.9	53.7 ± 6.1	53.1 ± 5.9	52.4 ± 5.7	52.2 ± 5.7	52.6 ± 5.7
Age at assessment of frailty, years	73.6 ± 6.1	74.5 ± 6.2	73.9 ± 6.1	73.2 ± 5.9	73.1 ± 5.9	73.3 ± 5.9
Gender						
Men	711 (47.0)	1429 (42.3)	744 (40.9)	665 (40.1)	1107 (41.0)	563 (37.2)
Women	801 (53.0)	1948 (57.7)	1076 (59.1)	994 (59.9)	1592 (59.0)	950 (62.8)
Dialect group						
Hokkien	702 (46.4)	1673 (49.5)	928 (51.0)	852 (51.4)	1347 (49.9)	834 (55.1)
Cantonese	810 (53.6)	1704 (50.5)	892 (49.0)	807 (48.6)	1352 (50.1)	679 (44.9)
Education						
No formal education	287 (19.0)	566 (16.8)	298 (16.4)	248 (14.9)	413 (15.3)	173 (11.4)
Primary school	705 (46.6)	1544 (45.7)	804 (44.2)	731 (44.1)	1135 (42.1)	594 (39.3)
Secondary school	428 (28.3)	1012 (30.0)	594 (32.6)	530 (32.0)	886 (32.8)	575 (38.0)
Diploma / University	92 (6.1)	255 (7.5)	124 (6.8)	50 (9.0)	265 (9.8)	171 (11.3)
Alcohol consumption						
Never	1168 (77.3)	2714 (80.4)	1458 (80.1)	1317 (79.4)	2119 (78.5)	1235 (81.6)
Monthly	140 (9.3)	279 (8.3)	149 (8.2)	155 (9.3)	253 (9.4)	131 (8.7)
Weekly	149 (9.9)	305 (9.0)	158 (8.7)	154 (9.3)	247 (9.1)	119 (7.9)
Daily	55 (3.6)	79 (2.3)	55 (3.0)	33 (2.0)	80 (3.0)	28 (1.8)
Smoking history						
Never smoker	1082 (71.6)	2629 (77.9)	1419 (78.0)	1342 (80.9)	2142 (79.4)	1305 (86.3)
Former smoker	158 (10.4)	294 (8.7)	176 (9.7)	131 (7.9)	245 (9.1)	111 (7.3)
Current smoker	272 (18.0)	454 (13.4)	225 (12.4)	186 (11.2)	312 (11.6)	97 (6.4)
Amount of sleep per day, hours	7.0 ± 1.0	7.0 ± 1.0	6.9 ± 1.0	7.0 ± 1.0	7.0 ± 1.0	6.9 ± 1.0
Weekly participation in vigorous work or strenuous sports	260 (17.2)	600 (17.8)	347 (19.1)	306 (18.4)	512 (19.0)	310 (20.5)
Amount of moderate activity per week						
None	1244 (82.3)	2539 (75.2)	1400 (76.9)	1251 (75.4)	2101 (77.8)	1077 (71.2)
0.5 to 3 h	189 (12.5)	537 (15.9)	263 (14.5)	277 (16.7)	414 (15.3)	295 (19.5)
≥ 4 h	79 (5.2)	301 (8.9)	157 (8.6)	131 (7.9)	184 (6.8)	141 (9.3)
Body mass index						
Underweight [< 18.5 kg/m^2^]	104 (6.9)	216 (6.4)	97 (5.3)	84 (5.1)	156 (5.8)	81 (5.4)
Normal [18.5 to 22.9 kg/m^2^]	612 (40.5)	1520 (45.0)	791 (43.5)	719 (43.3)	1217 (45.1)	695 (45.9)
Overweight [23.0 to 27.4 kg/m^2^]	646 (42.7)	1365 (40.4)	784 (43.1)	713 (43.0)	1085 (40.2)	612 (40.4)
Obese [≥ 27.5 kg/m^2^]	150 (9.9)	276 (8.2)	148 (8.1)	143 (8.6)	241 (8.9)	125 (8.3)
Hypertension	292 (19.3)	646 (19.1)	356 (19.6)	293 (17.7)	463 (17.2)	310 (20.5)
Coronary artery disease	45 (3.0)	80 (2.4)	30 (1.7)	27 (1.6)	29 (1.1)	35 (2.3)
Stroke	6 (0.4)	10 (0.3)	11 (0.6)	8 (0.5)	10 (0.4)	3 (0.2)
Diabetes	62 (4.1)	186 (5.5)	65 (3.6)	74 (4.5)	101 (3.7)	85 (5.6)
Cancer	25 (1.7)	74 (2.2)	25 (1.4)	30 (1.8)	34 (1.3)	34 (2.3)

^1^ DASH, Dietary Approaches to Stop Hypertension

^2^ Values were presented as mean (SD) or n (%) as appropriate

^3^ The characteristics of participants at baseline according to changes in diet quality were compared using one-way ANOVA for continuous variables, and chi-squared test for categorical variables. All *P*-values were < 0.05 except for distribution in changes in DASH groups by weekly participation in vigorous work or strenuous sports, body mass index categories, hypertension, and stroke

In the fully adjusted model (Models 2a and 2b), higher DASH scores at both baseline and follow-up 3 were associated with lower odds of physical frailty in a dose-response manner (*P*-trends: 0.025 and < 0.001 for baseline and follow-up 3, respectively). Compared to the lowest DASH score quartile, participants who were in the highest quartiles of DASH scores at baseline or at follow-up 3 had 15% (95% CI: 1–27%) and 51% (95% CI: 42–59%) lower odds of physical frailty, respectively (Table 2). The association between baseline DASH score quartiles and physical frailty were mainly driven by the statistically significant associations with weakness and exhaustion (Additional File 4); whilst the association between follow-up 3 DASH score quartiles and physical frailty were robust and statistically significant across all criteria of physical frailty (Additional File 5).

**Table 2:** Associations of DASH^1^ quartiles at baseline and follow-up 3 with physical frailty

	Quartile 1 OR (95% CI)	Quartile 2 OR (95% CI)	Quartile 3 OR (95% CI)	Quartile 4 OR (95% CI)	*P*_trend_
**DASH at baseline**					
Median Score (Range)	16 [7–17]	19 [18–20]	22 [21–23]	26 [24–35]	
Cases / N	461/2881	461/2955	435/3093	509/3651	
Model 1 ^2^	1.00	0.95 (0.82, 1.10)	0.83 (0.71, 0.96)	0.84 (0.72, 0.97)	0.010
Model 2a ^3^	1.00	0.95 (0.81, 1.10)	0.83 (0.71, 0.97)	0.85 (0.73, 0.99)	0.025
**DASH at follow-up 3**					
Median Score (Range)	15 [7–16]	19 [17–20]	22 [21–23]	26 [24–34]	
Cases / N	518/2485	615/3697	408/3026	325/3372	
Model 1 ^2^	1.00	0.77 (0.67, 0.89)	0.64 (0.55, 0.74)	0.47 (0.40, 0.55)	< 0.001
Model 2b ^4^	1.00	0.80 (0.69, 0.92)	0.63 (0.54, 0.74)	0.49 (0.41, 0.58)	< 0.001

^1^ DASH, Dietary Approaches to Stop Hypertension

^2^ Model 1: adjusted for age at physical tests (years), gender, dialect group (Hokkien, Cantonese), level of education (none, primary, secondary, A-level/university)

^3^ Model 2a: adjusted for Model 1 and the following variables at baseline: hypertension, coronary artery disease, stroke, diabetes, cancer, alcohol consumption (none, monthly, weekly, daily), smoking history (never, former, current), body mass index (< 18.5, 18.5–22.9, 23.0–27.4, 27.5 + kg/m^2^), amount of sleep per day (≤ 5, 6, 7, 8, 9 + hours), amount of strenuous sports per week (0, 0.5-1, 2–3, 4 + hours), amount of vigorous work per week (0, 0.5-3, 4–6, 7 + hours), amount of moderate activity per week (0, 0.5-1, 2–3, 4–6, 7 + hours), daily energy intake (kcal)

^4^ Model 2b: adjusted for Model 1 and baseline daily energy intake; the following variables at follow-up 2: alcohol consumption (none/monthly, weekly, daily) and amount of physical activity per week (0, 0.5-4, 4 + hours); and the following variables at follow-up 3: smoking history, body mass index, amount of sleep per day, and new cases of cancer, hypertension, coronary artery disease, stroke, diabetes

In the analysis by changes in DASH scores, compared with the “consistently low” group, participants in the “large increase” and “consistently high” groups had 22% (95% CI: 5–36%) and 26% (95% CI: 6–41%) lower odds of physical frailty, respectively (Table 3). These associations were mainly driven by the statistically significant associations with weakness and exhaustion (Additional File 6). Similarly, compared with participants who were in the lowest DASH score tertiles at baseline and follow-up 3 (reference group), participants who remained in the highest DASH score tertiles at both time points had the lowest odds of physical frailty (OR: 0.59, 95% CI: 0.48, 0.73). Additionally, significantly lower odds of physical frailty were observed in those who improved their DASH scores from the lowest (OR: 0.68, 95% CI: 0.51, 0.91) or second tertile at baseline (OR: 0.61, 95% CI: 0.48, 0.76) to the highest tertile at follow-up 3, compared with participants who were in the lowest DASH score tertiles at both time points (Table 4).

**Table 3:** Associations between changes in DASH^1^ scores from baseline to follow-up 3 and physical frailty

	Change in DASH scores categories					
	Consistently low (< 3% change and ≤ median DASH at both timepoints)	Large decrease (> 10% decrease)	Small decrease (3–10% decrease)	Small increase (3–10% increase)	Large increase (> 10% increase)	Consistently high (< 3% change and > median DASH at both timepoints)
	OR (95% CI)	OR (95% CI)	OR (95% CI)	OR (95% CI)	OR (95% CI)	OR (95% CI)
Cases / N	243/1512	628/3377	298/1820	217/1659	309/2699	171/1513
Model 1 ^2^	1.00	1.09 (0.92, 1.30)	1.01 (0.83, 1.23)	0.83 (0.68, 1.03)	0.73 (0.60, 0.88)	0.71 (0.57, 0.89)
Model 2 ^3^	1.00	1.13 (0.95, 1.35)	1.06 (0.87, 1.29)	0.86 (0.70, 1.06)	0.76 (0.63, 0.92)	0.74 (0.59, 0.92)
Model 3 ^4^	1.00	1.12 (0.94, 1.35)	1.10 (0.90, 1.35)	0.86 (0.70, 1.07)	0.78 (0.64, 0.95)	0.74 (0.59, 0.94)

^1^ DASH, Dietary Approaches to Stop Hypertension

^2^ Model 1: adjusted for age at physical tests (years), gender, dialect group (Hokkien, Cantonese), level of education (none, primary, secondary, A-level/university)

^3^ Model 2: adjusted for Model 1 and the following variables at baseline: hypertension, angina or heart attack, stroke, diabetes, cancer, alcohol consumption (none, monthly, weekly, daily), smoking history (never, former, current), body mass index (< 18.5, 18.5–22.9, 23.0–27.4, 27.5 + kg/m^2^), amount of sleep per day (≤ 5, 6, 7, 8, 9 + hours), amount of strenuous sports per week (0, 0.5-1, 2–3, 4 + hours), amount of vigorous work per week (0, 0.5-3, 4–6, 7 + hours), amount of moderate activity per week (0, 0.5-1, 2–3, 4–6, 7 + hours), daily energy intake (kcal)

^4^ Model 3: adjusted for Model 1 and the following variables at baseline: alcohol consumption, smoking history, body mass index, amount of sleep per day, weekly amount of strenuous sports, vigorous work, moderate activity, daily energy intake; the following variables at follow-up 2: alcohol consumption (none/monthly, weekly, daily) and amount of physical activity per week (0, 0.5-4, 4 + hours); and the following variables at follow-up 3: smoking history, body mass index, amount of sleep per day, and new cases of cancer, hypertension, coronary artery disease, stroke, diabetes

**Table 4:** Joint analyses of DASH1 tertiles at baseline and follow-up 3 with physical frailty2

	DASH tertiles at follow-up 3					
	Low (Median: 16, Range: 7–18)		Medium (Median: 21, Range: 19–22)		High (Median: 25, Range: 23–34)	
**DASH tertiles at baseline**	Cases / N	OR (95% CI)	Cases / N	OR (95% CI)	Cases / N	OR (95% CI)
Low (Median: 16, Range: 7–18)	343/1899	1.00	170/1152	0.86 (0.69, 1.07)	80/684	0.68 (0.51, 0.91)
Medium (Median: 21, Range: 19–22)	274/1412	1.04 (0.85, 1.27)	220/1458	0.88 (0.71, 1.08)	144/1388	0.61 (0.48, 0.76)
High (Median: 25, Range: 23–35)	180/862	1.08 (0.85, 1.35)	224/1437	0.84 (0.68, 1.04)	231/2288	0.59 (0.48, 0.73)

^1^ DASH, Dietary Approaches to Stop Hypertension

^2^ Models adjusted for age at physical tests (years), gender, dialect group (Hokkien, Cantonese), level of education (none, primary, secondary, A-level/university); the following variables at baseline: alcohol consumption, smoking history, body mass index, amount of sleep per day, weekly amount of strenuous sports, vigorous work, moderate activity, daily energy intake; the following variables at follow-up 2: alcohol consumption (none/monthly, weekly, daily) and amount of physical activity per week (0, 0.5-4, 4 + hours); and the following variables at follow-up 3: smoking history, body mass index, amount of sleep per day, and new cases of cancer, hypertension, coronary artery disease, stroke, diabetes

The associations between changes in diet quality and physical frailty did not significantly differ by age groups at baseline and gender (*P*-interaction > 0.05; Additional File 7). Finally, results remained the same in the analysis excluding participants who were 60 years old and above and/or had a history of hypertension, cardiovascular disease, diabetes, or cancer at baseline (Addiional File 8).

## Discussion

In this population-based cohort of middle-aged and older adults in Singapore, we observed that maintaining a higher diet quality since mid-life, defined using the DASH diet score, was prospectively associated with lower risk of physical frailty in late life. Further, substantially improving diet quality after midlife could still significantly lower the risk of physical frailty at late life.

Our finding of a high-quality diet at midlife associating with a lower risk of physical frailty in late life concurs with existing studies in both Western and Asian cohorts examining associations between dietary patterns at midlife and physical frailty at late life [11–16]. This finding is also reminiscent of our previous finding from the SCHS cohort showing that adherence to healthy dietary patterns at midlife was associated with a higher likelihood of healthy ageing (which included good physical functioning) at late life [28]. However, to the best of our knowledge, this is the first study in Asian populations to examine maintenance or changes in diet quality from mid to late life with physical frailty. We found that participants who had consistently high diet quality from mid to late life had the lowest risk of physical frailty. This finding is consistent with studies in Western populations showing that consistently higher adherence to a healthy diet (across a range of healthy eating indices such as the Mediterranean diet, the Alternative Healthy Eating Index, and the Dietary Inflammatory Index, or defined as higher adherence to national dietary guidelines) from midlife was associated with a lower risk of frailty [17, 18]. Hence, middle-aged and older adults should be encouraged to maintain high diet quality throughout middle and older adulthood to achieve the most favourable outcome in physical function and capacity.

More importantly, our study showed that an improvement in diet quality could still lower the risk of physical frailty. A large improvement (> 10% change in DASH scores), achieved by a 1-quintile change in three to four DASH components (e.g. increasing 1 serving of vegetables, 0.5 serving of fruit, 0.5 serving of nuts and legumes, and decreasing 0.2 serving of sugar-sweetened beverages per day based on average intakes in our cohort), could lower the risk of physical frailty. Alternatively, if the participants had a moderate diet quality (middle DASH tertile) at baseline, an improvement to the highest DASH tertile at follow-up 3 was able to lower their risk of physical frailty. Furthermore, even if the participants had started out with a poor diet quality at baseline (lowest DASH tertile), by substantially improving their diet quality to the highest DASH tertile at follow-up 3, they could still achieve a lower risk of frailty compared to their counterparts who maintained a poor diet quality throughout. Improving from lowest to highest tertile can be achieved by increasing 2 servings of vegetables, 0.5 serving of fruit, 0.5 serving of nuts and legumes, and decreasing 0.2 serving of sugar-sweetened beverages per day based on our cohort data. Our findings suggest that dietary interventions implemented even after midlife remains important in preventing or delaying physical frailty in late life. Taken together, midlife signifies an opportune time for interventions or health promotion to encourage adoption of healthy or high-quality dietary patterns to prevent or delay onset of age-related conditions that includes physical frailty in late life.

There are several biologically plausible explanations to the role of the DASH diet in lowering risk of physical frailty. First, higher DASH scores reflect higher adherence to a healthy dietary pattern which has been related to lower risks of several age-related chronic conditions such as cardiovascular diseases [29], type-2 diabetes [30], cancer [31] and sarcopenia [32] that could increase the risk of physical frailty. Second, the DASH diet advocates higher consumption of fruit and vegetables which are rich in antioxidants shown to decrease oxidative stress related to the pathophysiology of frailty [10]. Third, emerging evidence suggest that higher intake of dairy products (a component of the DASH diet) may lower risk of physical frailty [10], because dairy products are important contributors of protein and vitamins D and B12 intakes, which have been shown to have beneficial effects on muscle health [4, 10].

Among the four criteria for physical frailty, we found that a consistently high or improved diet quality from mid to late life was significantly associated with lower weakness and exhaustion. The observed association between diet quality and weakness in this study is expected, as several studies have shown that adhering to a high-quality diet was associated with increased handgrip strength [33, 34] – used to define weakness in our study. It is unclear how having a high diet quality influences exhaustion, but several dietary interventions aimed to increase adherence to a healthy diet (e.g., the Mediterranean diet) have been effective in reducing fatigue [35]. We speculate that maintaining a high diet quality or improving diet quality contributes to meeting energy and nutritional requirements, and thus may prevent catabolism with fatigue as a consequential symptom [36].

The strengths of our study included the prospective design, long follow-up period spanning mid to late life, and the repeated measures of dietary intake. Several limitations should be noted. First, reverse causation was possible as we could not identify and exclude participants with physical frailty at baseline because we did not conduct the relevant assessments. Nonetheless, the likelihood of being physically frail at midlife would have been low. Furthermore, in our sensitivity analysis, we excluded participants who were more likely to have been physically frail at baseline (aged ≥ 60 years and/or with a baseline history of hypertension, cardiovascular disease, diabetes, or cancer), and our effect estimates remained essentially unchanged. Second, the risk estimates may have been underestimated due to survivorship bias. This is because participants who remained in the cohort were more likely to be those who had a healthier lifestyle and fewer comorbidities [37], and thus at a lower risk for physical frailty. Third, the dietary questionnaire used at follow-up 3 differed from baseline, as such, diet quality across the time points may not be directly comparable and may affect the reliability of the observed associations. However, we had ensured that the same food items were used for the calculation of the DASH scores at both timepoints. We had also examined changes in DASH score tertiles in addition to changes in absolute scores, which would minimise systematic errors resulting from differences in dietary questionnaires, and shown similar results with both approaches. The exclusion of the sodium component from the calculation of DASH score may not comprehensively reflect the DASH diet; however, we had previously demonstrated that this DASH score, without the sodium component, remained reliable in capturing overall diet quality, as this score had similar associations with cardiovascular disease risk factors, in terms of direction and magnitude, when compared with other indices of diet quality (e.g. alternate Mediterranean Diet score) [19]. We also acknowledge the limitation of self-reported dietary intake, which is subjected to recall error and social desirability bias, but in a prospective design like our study, this would likely result in nondifferential misclassification leading to an underestimation of risk estimates. Fourth, we utilized a modified version of the CHS frailty phenotype in this study that contained only four of the original five criteria as we did not have data on physical activity level. In doing so, we adopted a conservative definition of frailty as meeting at least two of four available criteria, and thus may have misclassified pre-frail subjects as frail, leading to an over-estimation of the prevalence of frailty. Nonetheless, as previously reported [24], the prevalence of physical frailty among women aged 70–79 years in our cohort (14.4% frail) was comparable to that of three other cohorts that had used the original CHS frailty phenotype [2, 38, 39]. Additionally, we acknowledge some limitations in the other criteria of our modified frailty phenotype. For the criterion of weight loss, we used weight at follow-up 2, which was reported average 7 years prior, to calculate participants’ change in body weight at follow-up 3. This may not, as in the original definition, have strictly reflected unintentional weight loss in the year prior to the assessment of frailty; nevertheless, in older adults, studies have shown that self-reported weight loss of ≥ 5%, even when compared to weight 10 years ago, was associated with an increased risk of frailty [40]. For the criterion of slowness, we used the TUG test in place of gait speed. Compared to gait speed, the TUG test involves additional functional movements such as standing, turning, and sitting; nonetheless, studies have observed high correlations between performance on both tests [41, 42], and the TUG test itself has been shown to be a sensitive and specific discriminator for frailty in older adults [43].

## Conclusions

In conclusion, our study found that maintaining a high-quality diet or substantially improving diet quality from mid to late life was associated with a lower risk of physical frailty in a cohort of older Chinese adults. These findings highlight the need to support middle-aged adults to maintain their diet quality until old age, or to substantially improve their diet quality after midlife to prevent or delay physical frailty.

### Electronic supplementary material

Below is the link to the electronic supplementary material.

Supplementary Material 1

Supplementary Material 2

## Data Availability

Data described in the manuscript, code book, and analytic code will be made available upon request pending approval by the senior author W-PK.

## Abbreviations

BMI: Body Mass Index
CHS: Cardiovascular Health Study
DASH: Dietary Approaches to Stop Hypertension
FFQ: Food Frequency Questionnaire
SCHS: Singapore Chinese Health Study
TUG: Timed Up-and-Go
