# Supplementary material for: Diet quality from mid to late life and its association with physical frailty in late life in a cohort of Chinese adults
Source: Nutr J. 2024 Jun 4;23:57. doi: 10.1186/s12937-024-00964-y (PMC11149349; doi:10.1186/s12937-024-00964-y)
Supplement: Supplementary file 1 — Supplementary Material 1 [file 12937_2024_964_MOESM1_ESM.docx]

Participants who completed baseline interviews including the FFQ

(*n* = 63,257)

Participants who completed assessment of physical frailty at follow-up 3

(*n* = 12,580)

Participants who completed dietary screener at follow-up 3

(*n* = 14,802)

Participants who completed follow-up 2 interviews and self-reported weight (relevant to this study)

(*n* = 39,258)

**Additional File 2**: Participant flowchart for the analysis examining changes in diet quality and physical frailty in the Singapore Chinese Health Study. FFQ, Food Frequency Questionnaire.

**Additional File 3:** Characteristics of study participants at follow-up 3 (unless otherwise specified) by change in DASH^1^ scores^2^

|  | Consistently low  (<3% change and ≤median DASH at both timepoints) | Large decrease  (>10% decrease) | Small decrease  (3-10% decrease) | Small increase  (3-10% increase) | Large increase  (>10% increase) | Consistently high  (<3% change and >median DASH at both timepoints) | *P* |
| --- | --- | --- | --- | --- | --- | --- | --- |
| Alcohol consumption at follow-up 2 |  |  |  |  |  |  |  |
| Never/Monthly | 1338 (88.5) | 3075 (91.1) | 1650 (90.7) | 1509 (91.0) | 2439 (90.4) | 1377 (91.0) | 0.021 |
| Weekly | 121 (8.0) | 213 (6.3) | 124 (6.8) | 117 (7.0) | 192 (7.1) | 114 (7.5) |  |
| Daily | 53 (3.5) | 89 (2.6) | 46 (2.5) | 33 (2.0) | 68 (2.5) | 22 (1.5) |  |
| Physical activity^3^ at follow-up 2 |  |  |  |  |  |  |  |
| None | 124 (8.2) | 232 (6.9) | 98 (5.4) | 104 (6.3) | 143 (5.3) | 71 (4.7) | <0.001 |
| 0.5-4 hours | 243 (16.1) | 461 (13.7) | 265 (14.6) | 235 (14.2) | 378 (14.0) | 171 (11.3) |  |
| 4+ hours | 1145 (75.7) | 2684 (79.5) | 1457 (80.0) | 1320 (79.6) | 2178 (80.7) | 1271 (84.0) |  |
| Smoking history |  |  |  |  |  |  |  |
| Never smoker | 1057 (69.9) | 2542 (75.3) | 1385 (76.1) | 1306 (78.7) | 2087 (77.3) | 1270 (83.9) | <0.001 |
| Former smoker | 277 (18.3) | 524 (15.5) | 278 (15.3) | 245 (14.8) | 447 (16.6) | 196 (13.0) |  |
| Current smoker | 178 (11.8) | 311 (9.2) | 157 (8.6) | 108 (6.5) | 165 (6.1) | 47 (3.1) |  |
| Body mass index |  |  |  |  |  |  |  |
| Underweight [<18.5 kg/m^2^] | 584 (38.6) | 1438 (42.5) | 743 (40.8) | 691 (41.6) | 1149 (42.6) | 635 (42.0) | <0.001 |
| Normal [18.5-22.9 kg/m^2^] | 140 (9.3) | 363 (10.8) | 153 (8.4) | 137 (8.3) | 239 (8.9) | 133 (8.8) |  |
| Overweight [23.0 to 27.4 kg/m^2^] | 557 (36.8) | 1213 (35.9) | 723 (39.7) | 628 (37.9) | 984 (36.4) | 586 (38.7) |  |
| Obese [≥ 27.5 kg/m^2^] | 231 (15.3) | 363 (10.8) | 201 (11.1) | 203 (12.2) | 327 (12.1) | 159 (10.5) |  |
| Amount of sleep per day, hours | 6.8 ± 1.3 | 6.8 ± 1.3 | 6.7 ± 1.3 | 6.7 ± 1.2 | 6.7 ± 1.2 | 6.7 ± 1.2 | 0.154 |
| Hypertension | 679 (44.9) | 1519 (45.0) | 783 (43.0) | 728 (43.9) | 1193 (44.2) | 627 (41.4) | 0.077 |
| Heart attack | 153 (10.1) | 338 (10.0) | 179 (9.8) | 157 (9.5) | 231 (8.6) | 161 (10.6) | <0.001 |
| Stroke | 84 (5.6) | 168 (5.0) | 66 (3.6) | 80 (4.8) | 103 (3.8) | 53 (3.5) | 0.024 |
| Diabetes | 285 (18.8) | 537 (15.9) | 300 (16.5) | 305 (18.4) | 541 (20.0) | 261 (17.2) | <0.001 |
| Cancer | 87 (5.7) | 177 (5.2) | 91 (5.0) | 94 (5.7) | 159 (5.9) | 96 (6.3) | 0.116 |

^1^ DASH, Dietary Approaches to Stop Hypertension

^2^ Values were presented as n (%) or mean ± SD.

^3^ Hours per week spent on moderate activities, strenuous sports, and vigorous work.

**Additional File 4**: Associations between DASH^1^ quartiles at baseline and the individual physical frailty criteria

|  | **DASH quartiles at baseline** | | | | *P*_trend_ |
| --- | --- | --- | --- | --- | --- |
|  | Quartile 1 | Quartile 2 | Quartile 3 | Quartile 4 |  |
| **Frailty criteria** | | | | |  |
| Slowness (TUG test time in the slowest sex-specific quintile) | | | | |  |
| Cases / N | 482/2881 | 465/2955 | 467/3093 | 553/3651 |  |
| OR (95% CI) ^2^ | 1.00 | 0.91 (0.78, 1.07) | 0.88 (0.75, 1.03) | 0.94 (0.80, 1.11) | 0.522 |
| Weakness (handgrip strength in the weakest sex-specific quintile) | | | | |  |
| Cases / N | 603/2881 | 574/2955 | 568/3093 | 612/3651 |  |
| OR (95% CI) ^2^ | 1.00 | 0.86 (0.75, 0.98) | 0.79 (0.68, 0.90) | 0.69 (0.59, 0.79) | <0.001 |
| Weight loss (lost ≥10% of self-reported body weight since the previous follow-up interview) | | | | |  |
| Cases / N | 344/2881 | 346/2955 | 348/3093 | 382/3651 |  |
| OR (95% CI) ^2^ | 1.00 | 0.96 (0.82, 1.13) | 0.92 (0.78, 1.09) | 0.86 (0.72, 1.02) | 0.064 |
| Exhaustion (answered “No” to the question: “Do you feel full of energy?”) | | | | |  |
| Cases / N | 536/2881 | 563/2955 | 545/3093 | 577/3651 |  |
| OR (95% CI) ^2^ | 1.00 | 1.04 (0.91, 1.19) | 0.93 (0.81, 1.07) | 0.83 (0.72, 0.95) | 0.001 |

^1^ DASH, Dietary Approaches to Stop Hypertension; TUG, timed up-and-go.

^2^ Models adjusted for age at physical tests (years), gender, dialect group (Hokkien, Cantonese), level of education (none, primary, secondary, A-level/university), and baseline hypertension, angina or heart attack, stroke, diabetes, cancer, alcohol consumption (none, monthly, weekly, daily), smoking history (never, former, current), body mass index (<18.5, 18.5-22.9, 23.0-27.4, 27.5+ kg/m^2^), amount of sleep per day (≤5, 6, 7, 8, 9+ hours), amount of strenuous sports per week (0, 0.5-1, 2-3, 4+ hours), amount of vigorous work per week (0, 0.5-3, 4-6, 7+ hours), amount of moderate activity per week (0, 0.5-1, 2-3, 4-6, 7+ hours), daily energy intake (kcal).

**Additional File 5**: Associations between DASH^1^ quartiles at follow-up 3 and the individual physical frailty criteria.

|  | **DASH quartiles at follow-up 3** | | | | *P*_trend_ |
| --- | --- | --- | --- | --- | --- |
|  | Quartile 1 | Quartile 2 | Quartile 3 | Quartile 4 |  |
| **Frailty criteria** | | | | |  |
| Slowness (TUG test time in the slowest sex-specific quintile) | | | | |  |
| Cases / N | 489/2485 | 631/3697 | 442/3026 | 405/3372 |  |
| OR (95% CI) ^2^ | 1.00 | 0.92 (0.80, 1.06) | 0.81 (0.69, 0.95) | 0.80 (0.67, 0.94) | 0.003 |
| Weakness (handgrip strength in the weakest sex-specific quintile) | | | | |  |
| Cases / N | 621/2882 | 766/2956 | 518/3094 | 452/3651 |  |
| OR (95% CI) ^2^ | 1.00 | 0.77 (0.68, 0.88) | 0.62 (0.54, 0.71) | 0.48 (0.42, 0.56) | <0.001 |
| Weight loss (lost ≥10% of self-reported body weight since the previous follow-up interview) | | | | |  |
| Cases / N | 337/2882 | 456/2956 | 325/3094 | 302/3651 |  |
| OR (95% CI) ^2^ | 1.00 | 0.92 (0.79, 1.07) | 0.81 (0.68, 0.95) | 0.71 (0.60, 0.85) | <0.001 |
| Exhaustion (answered “No” to the question: “Do you feel full of energy?”) | | | | |  |
| Cases / N | 538/2882 | 698/2956 | 535/3094 | 450/3651 |  |
| OR (95% CI) ^2^ | 1.00 | 0.84 (0.74, 0.96) | 0.77 (0.67, 0.89) | 0.56 (0.48, 0.65) | <0.001 |

^1^ DASH, Dietary Approaches to Stop Hypertension; TUG, timed up-and-go.

^2^ Models adjusted for age at physical tests (years), gender, dialect group (Hokkien, Cantonese), level of education (none, primary, secondary, A-level/university), baseline energy intake, follow-up 2 variables of alcohol consumption and amount of physical activity per week; and follow-up 3 variables of smoking history, body mass index, amount of sleep per day (≤5, 6, 7, 8, 9+ hours), and new cases of hypertension, coronary artery disease, stroke, diabetes.

**Additional File 6:** Associations between changes in DASH^1^ scores from baseline to follow-up 3 and individual physical frailty criteria.

|  | **Change in DASH scores categories** | | | | | |
| --- | --- | --- | --- | --- | --- | --- |
|  | Consistently low  (<3% change and ≤median DASH at both timepoints) | Large decrease  (>10% decrease) | Small decrease  (3-10% decrease) | Small increase  (3-10% increase) | Large increase  (>10% increase) | Consistently high  (<3% change and >median DASH at both timepoints) |
| **Frailty criteria** | | | | | |  |
| Slowness (TUG test time in the slowest sex-specific quintile) | | | | | |  |
| Cases / N | 252/1512 | 615/3377 | 311/1820 | 249/1659 | 347/2699 | 193/1513 |
| OR (95% CI) ^2^ | 1.00 | 1.05 (0.87, 1.27) | 1.12 (0.91, 1.38) | 1.03 (0.83, 1.28) | 0.89 (0.73, 1.08) | 0.89 (0.71, 1.13) |
| Weakness (handgrip strength in the weakest sex-specific quintile) | | | | | |  |
| Cases / N | 307/1512 | 730/3377 | 379/1820 | 276/1659 | 419/2699 | 246/1513 |
| OR (95% CI) ^2^ | 1.00 | 0.99 (0.84, 1.16) | 1.05 (0.88, 1.26) | 0.82 (0.68, 0.99) | 0.77 (0.65, 0.92) | 0.79 (0.65, 0.97) |
| Weight loss (lost ≥10% of self-reported body weight since the previous follow-up interview) | | | | | |  |
| Cases / N | 180/1512 | 448/3377 | 200/1820 | 158/1659 | 290/2699 | 144/1513 |
| OR (95% CI) ^2^ | 1.00 | 1.03 (0.84, 1.27) | 0.92 (0.73, 1.16) | 0.80 (0.62, 1.02) | 0.94 (0.76, 1.17) | 0.80 (0.62, 1.03) |
| Exhaustion (answered “No” to the question: “Do you feel full of energy?”) | | | | | |  |
| Cases / N | 302/1512 | 678/3377 | 301/1820 | 269/1659 | 436/2699 | 235/1513 |
| OR (95% CI) ^2^ | 1.00 | 1.03 (0.88, 1.21) | 0.83 (0.69, 1.00) | 0.80 (0.66, 0.96) | 0.81 (0.69, 0.96) | 0.77 (0.63, 0.94) |

^1^ DASH, Dietary Approaches to Stop Hypertension; TUG, timed up-and-go.

^2^ Models adjusted for age at physical tests (years), sex, dialect group (Hokkien, Cantonese), level of education (none, primary, secondary, A-level/university); the following variables at baseline: alcohol consumption, smoking history, body mass index, amount of sleep per day, weekly amount of strenuous sports, vigorous work, moderate activity, daily energy intake; the following variables at follow-up 2: alcohol consumption (none/monthly, weekly, daily) and amount of physical activity per week (0, 0.5-4, 4+hours); and the following variables at follow-up 3: smoking history, body mass index, amount of sleep per day, and new cases of cancer, hypertension, coronary artery disease, stroke, diabetes

**Additional File 7:** Associations between changes in DASH^1^ from baseline to follow-up 3 and physical frailty, stratified by age group at baseline interviews and sex.

|  | **DASH change groups** | | | | | |
| --- | --- | --- | --- | --- | --- | --- |
|  | Consistently low  (<3% change and ≤median DASH at both timepoints) | Large decrease  (>10% decrease) | Small decrease  (3-10% decrease) | Small increase  (3-10% increase) | Large increase  (>10% increase) | Consistently high  (<3% change and >median DASH at both timepoints) |
| **Stratified by age group at baseline interviews** | | | | | | |
| < 55 years |  |  |  |  |  |  |
| Cases / N | 88/877 | 229/2112 | 118/1202 | 99/1150 | 131/1983 | 63/1050 |
| OR (95% CI) ^2^ | 1.00 | 1.29 (0.99, 1.70) | 1.33 (0.99, 1.80) | 1.00 (0.73, 1.38) | 0.83 (0.62, 1.11) | 0.74 (0.52, 1.05) |
| ≥ 55 years |  |  |  |  |  |  |
| Cases / N | 137/446 | 419/1335 | 163/605 | 123/479 | 173/757 | 123/521 |
| OR (95% CI) ^2^ | 1.00 | 1.00 (0.78, 1.28) | 0.94 (0.71, 1.25) | 0.76 (0.56, 1.03) | 0.75 (0.57, 0.98) | 0.76 (0.55, 1.04) |
| *P*-interaction |  | 0.266 | 0.155 | 0.236 | 0.519 | 0.825 |
| **Stratified by gender** | | | | | | |
| Men |  |  |  |  |  |  |
| Cases / N | 97/620 | 275/1431 | 120/760 | 101/667 | 128/1130 | 51/611 |
| OR (95% CI) ^2^ | 1.00 | 1.28 (0.97, 1.70) | 1.28 (0.93, 1.76) | 0.99 (0.71, 1.39) | 0.94 (0.69, 1.28) | 0.69 (0.46, 1.03) |
| Women |  |  |  |  |  |  |
| Cases / N | 128/703 | 373/2016 | 161/1047 | 121/962 | 176/1610 | 135/1023 |
| OR (95% CI) ^2^ | 1.00 | 1.02 (0.80, 1.30) | 0.96 (0.73, 1.26) | 0.78 (0.59, 1.04) | 0.67 (0.51, 0.87) | 0.74 (0.55, 0.99) |
| *P*-interaction |  | 0.186 | 0.189 | 0.292 | 0.131 | 0.712 |

^1^ DASH, Dietary Approaches to Stop Hypertension

^2^ Models adjusted for age at physical tests (years), gender, dialect group (Hokkien, Cantonese), level of education (none, primary, secondary, A-level/university), the following variables at baseline: alcohol consumption (none, monthly, weekly, daily), smoking history (never, former, current), body mass index (<18.5, 18.5-22.9, 23.0-27.4, 27.5+ kg/m^2^), amount of sleep per day (≤5, 6, 7, 8, 9+ hours), amount of strenuous sports per week (0, 0.5-1, 2-3, 4+ hours), amount of vigorous work per week (0, 0.5-3, 4-6, 7+ hours), amount of moderate activity per week (0, 0.5-1, 2-3, 4-6, 7+ hours), daily energy intake (kcal), the following variables at follow-up 2: alcohol consumption and amount of physical activity per week, and the following variables at follow-up: smoking history, body mass index, amount of sleep per day (≤5, 6, 7, 8, 9+ hours), and new cases of cancer, hypertension, coronary artery disease, stroke, diabetes

**Additional File 8:** Associations between changes in DASH^1^ scores from baseline to follow-up 3 and physical frailty, among participants who were <60 years old and free from hypertension, cardiovascular disease, diabetes, and cancer at baseline

|  | **Change in DASH scores categories** | | | | | |
| --- | --- | --- | --- | --- | --- | --- |
|  | Consistently low  (<3% change and ≤median DASH at both timepoints) | Large decrease  (>10% decrease) | Small decrease  (3-10% decrease) | Small increase  (3-10% increase) | Large increase  (>10% increase) | Consistently high  (<3% change and >median DASH at both timepoints) |
| Cases / N | 105/1017 | 261/2138 | 131/1221 | 118/1157 | 140/1931 | 66/1010 |
| OR (95% CI) ^2^ | 1.00 | 1.19 (0.92, 1.54) | 1.17 (0.87, 1.55) | 1.09 (0.81, 1.46) | 0.74 (0.57, 0.99) | 0.73 (0.51, 1.01) |

^1^ DASH, Dietary Approaches to Stop Hypertension

^2^ Model adjusted for age at physical tests (years), sex, dialect group (Hokkien, Cantonese), level of education (none, primary, secondary, A-level/university); the following variables at baseline: alcohol consumption (none, monthly, weekly, daily), smoking history (never, former, current), body mass index (<18.5, 18.5-22.9, 23.0-27.4, 27.5+ kg/m^2^), amount of sleep per day (≤5, 6, 7, 8, 9+ hours), amount of strenuous sports per week (0, 0.5-1, 2-3, 4+ hours), amount of vigorous work per week (0, 0.5-3, 4-6, 7+ hours), amount of moderate activity per week (0, 0.5-1, 2-3, 4-6, 7+ hours), daily energy intake (kcal); the following variables at follow-up 2: alcohol consumption and amount of physical activity per week (0, 0.5-4, 4+hours); and the following variables at follow-up 3: smoking history, body mass index, amount of sleep per day, and new cases of cancer, hypertension, coronary artery disease, stroke, diabetes
